# Successful lichen translocation on disturbed gypsum areas: A test with adhesives to promote the recovery of biological soil crusts

**DOI:** 10.1038/srep45606

**Published:** 2017-04-03

**Authors:** M. Ballesteros, J. Ayerbe, M. Casares, E. M. Cañadas, J. Lorite

**Affiliations:** 1 Department of Botany, Faculty of Sciences, University of Granada, Campus de Fuentenueva s/n, Granada, 18071, Spain; 2 iEcolab. Interuniversitary Institute for Earth System Research (IISTA) - Universidad de Granada, Av. del Mediterráneo, 18006, Granada, Spain

## Abstract

The loss of biological soil crusts represents a challenge for the restoration of disturbed environments, specifically in particular substrates hosting unique lichen communities. However, the recovery of lichen species affected by mining is rarely addressed in restoration projects. Here, we evaluate the translocation of *Diploschistes diacapsis,* a representative species of gypsum lichen communities affected by quarrying. We tested how a selection of adhesives could improve thallus attachment to the substrate and affect lichen vitality (as CO_2_ exchange and fluorescence) in rainfall-simulation and field experiments. Treatments included: white glue, water, hydroseeding stabiliser, gum arabic, synthetic resin, and a control with no adhesive. Attachment differed only in the field, where white glue and water performed best. Adhesives altered CO_2_ exchange and fluorescence yield. Notably, wet spoils allowed thalli to bind to the substrate after drying, revealing as the most suitable option for translocation. The satisfactory results applying water on gypsum spoils are encouraging to test this methodology with other lichen species. Implementing these measures in restoration projects would be relatively easy and cost-effective. It would help not only to recover lichen species in the disturbed areas but also to take advantage of an extremely valuable biological material that otherwise would be lost.

Biological soil crusts (BSCs), consisting of complex associations of lichens, mosses, algae, cyanobacteria, and other organisms are remarkable components of dryland ecosystems worldwide^1^. They play a critical role in their structure and function, acting on soil stability, biogeochemical cycling, and plant establishment^1^,^2^,^3^. These crusts are particularly notable in gypsum ecosystems, and are normally dominated by lichens^1^,^4^,^5^. They have a high conservation value due to their potential to form covers (sometimes >80%), their function, and their diversity^6^,^7^,^8^. However, large extensions covered by BSCs are disturbed by human activities^9^,^10^, quarrying inflicting particularly severe damage^6^,^11^. Gypsum is a mineral in global demand^12^ and its quarrying inevitably damages BSCs and their habitat. Despite that mining companies are obligated to conduct environmental restoration (e.g. geomorphology and plant cover), BSCs are normally ignored. Limitations such as the slow growth of their components, low reproduction rates, the destruction of propagules and habitat together with the lack of a clear restoration methodology make the recovery of BSCs especially challenging^3^,^10^,^13^,^14^.

Few studies have addressed the assisted recovery of BSCs. Practice to enhance BSC establishment include soil stabilisation, resource augmentation, and inoculation-based techniques^10^. Inoculation is the best-studied and consists of adding cultured or salvaged BSC components to increase propagule availability in the disturbed area^14^,^15^,^16^,^17^. The recovery of cyanobacteria and algae has been achieved in gypsum BSCs over the short term^14^,^15^,^16^. However, the recovery of later successional components such as lichens can be extremely slow unless these are translocated^9^,^14^,^15^. Lichen translocation can be particularly advisable to accelerate species recolonisation in the disturbed area, but the loss of thalli due to environmental factors (e.g. wind, rain-storms) can arrest or greatly delay establishment^9^,^18^.

The attachment of propagules to the substrate is key for full lichen development (e.g. nutrient and moisture intake, hyphae formation, and favourable reproduction)^19^,^20^ and acts as one of the main ecological filters for colonisation and recovery^21^. The first step for propagules is to attach to the substrate in an entirely physical process^20^. Thereafter, propagules start to grow hyphae and actively attach to the substrate^20^. Otherwise, dispersed propagules remain erratic at risk of being removed off by wind^22^ or water^23^ and lost. Thus, keeping thalli in place would be useful in reducing their loss, favouring establishment, and eventually augmenting the source of propagules over time to hasten BSC recovery. Accordingly, a range of adhesives has been used previously for lichen translocation on different substrates (see Smith^24^ for a review), terricolous habitats being especially challenging. The aim of our study is to assess thallus translocation for restoration using a representative species of gypsum lichen communities [*Diploschistes diacapsis* (Ach.) Lumbsch]. We tested whether the application of various adhesives could improve thallus attachment onto gypsum mine spoil without compromising their vitality. The results may be helpful for future restoration programs to recover BSCs in disturbed gypsum habitats.

## Material and Methods

### Site description

**Figure 1:**
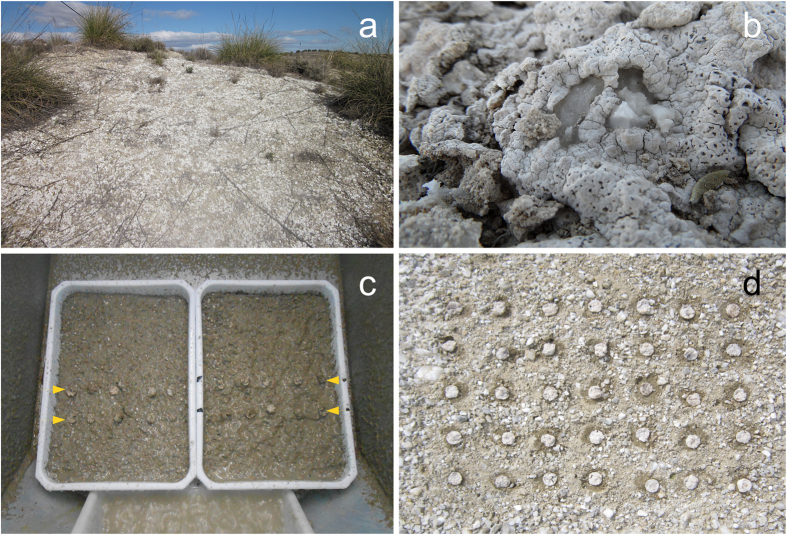
(**a**) Soil crust community on gypsum soil dominated by the lichen *Diploschistes diacapsis* in a Mediterranean gypsum shrub community in Granada, Spain. (**b**) *D. diacapsis* growing on gypsum soil. (**c** and **d**) Thalli of *D. diacapsis* used in our experiments to test for the effect of various adhesives on thallus attachment to gypsum spoil; (**c**) in trays during the rainfall-simulation (see arrows), and (**d**) in one of the plots when the field experiment was set up in March 2014.

The field work was conducted in an experimental area next to an active quarry in Escúzar, Granada, SE Spain (37°2′N, 3°45′W) at 950 m asl. The climate is continental Mediterranean, with relatively cold winters, hot summers, and four months of water deficit. The mean annual temperature is 15.1 °C, with an average monthly minimum temperature in January of 7.6 °C and maximum of 24.2 °C in August. The annual rainfall, occurring mainly in winter, averages 420 mm. The area is in the Neogene sedimentary basin of Granada, where the dominant substrates are lime and gypsum deposited in the late Miocene, the latter in combination with marls^25^. The predominant soils where gypsum crops out are Gypsiric Leptosols^26^,^27^. The vegetation of the area is a mosaic of fields with cereal crops and olive and almond orchards (*Olea europaea* L. and *Prunus dulcis* D.A. Webb.), and scattered patches of native scrub described as 1520, “Iberian gypsum vegetation, *Gypsophiletalia*”^28^. Open areas between native plants are often colonised by a well-developed biocrust community characterised by lichens such as *D. diacapsis, Acarospora placodiiformis, A. nodulosa* var. *reagens, Buellia zoharyi, Squamarina lentigera, S. cartilaginea, F. desertorum, F. fulgens, F. poeltii, F. subbracteata, Psora decipiens,* and *P. saviczii* (Fig. 1a, Supplementary Table S1).

### Study species

*D. diacapsis* (Fig. 1b), is a sub-cosmopolitan terricolous crustose lichen species occurring mainly on calcareous and gypsaceous soils in open xeric habitats in Mediterranean and arid climates^1^,^29^,^30^. This lichen species has a thick thallus (1–3 mm) of variable aspect, verrucose-areolate with urceolate apothecia and white pruina^31^. It is one of the most frequent lichens in gypsum BSCs in the Iberian Peninsula, normally reaching the greatest cover and biomass (~40% cover^4^; ~55% cover and >80% biomass in the study area according Ibarz^32^). Having a central role in gypsum communities, it acts as host to numerous fungi and lichen species^33^. *D. diacapsis* was selected as a model species for its dominance at the study site and for its similar ecology and morphology with respect to other crust-forming lichens in gypsum habitats (e.g. *Acarospora, Buellia, Squamarina, Lecidea*)^34^.

### Common set up for rainfall-simulation and field experiments

#### Thallus collection

In March 2014, thalli were detached at the study site from the parent gypsum substrate and collected from shrub interspaces in the area where quarrying was scheduled. The samples were taken immediately to the laboratory in paper bags. Thalli were soaked in tap water (for 5–10 min), cleaned and die cut into disks of 15 mm to obtain homogeneous experimental units. Thalli were transferred to be used, respectively, in rainfall-simulation and field experiments within the next 24 hours.

#### Substrate used

Gypsum spoil was assayed as the recipient substrate for translocation (properties in Supplementary Table S2). It is a by-product of gypsum quarrying normally used to fill quarry pits and reshape the landscape after quarrying. Pilot studies have confirmed its suitability to conduct gypsum vegetation recovery^35^,^36^. This material, often remaining in the quarried areas, is a suitable substrate to test lichen and adhesive performance.

#### Adhesive treatments

Several commercial natural or synthetic adhesives were applied to thalli in rainfall-simulation and field experiments. These included: (1) white glue (G): wood and craft white glue consisting of polyvinyl acetate (Wolfpack, A Forged Tool S.A.); (2) hydroseeding stabiliser (HS): synthetic polyacrylamide polymer (Bonterra Ibérica S.L.) as powder dissolved in water at 5 g/L; (3) gum arabic (GA): a complex of glycoproteins and polysaccharides derived from *Acacia* tree exudates (Prager, Orita S.A.); (4) synthetic resin (SR): contact adhesive polymeric glue (Kollant, Impex Europa S.L.); and (5) water (W): wetting spoil with water. A control treatment (C) where no adhesive was applied to thalli was used in both experiments. The adhesives were used at their commercial concentrations.

#### Rainfall-simulation experiment

We tested the effect of adhesives on thallus attachment under controlled rainfall and slope conditions in a rainfall-simulation experiment recreating the effect of two separate disturbing rain events. In March 2014, 30 plastic trays (35 × 25 × 5 cm) were perforated to allow drainage, filled with gypsum spoil (sieved at 0.5 cm; approx. 2.5 Kg), watered, and left to dry in order for the spoil to gain cohesion. Ten thalli were transferred to each of five replicate trays after applying one of the six adhesive treatments (2 ml with a 200-ml syringe) to the lower surface of the thallus (i.e. closest side to substrate), except for the water treatment, which was applied by spraying tap water (~100 ml) to moisten the tray substrate (10 thalli × 5 replicates × 6 adhesive treatments = 300 thalli). Thalli were placed in two lengthwise parallel rows (5 each) in the middle of the tray (Fig. 1c), using a quadrat with a 5 × 5 cm grid, which also served to monitor detachment after rainfall simulations. Simulations were conducted in a greenhouse, using a rainfall simulator (modified from Fernández-Gálvez *et al*.^37^), consisting of a drop-forming chamber (controlled by a pump connected to a water reservoir) on top of a structure (2 m high) that housed two random trays at the same time tilted for a 25° slope (near the angle of rest of the spoil material to simulate an extreme situation). We conducted two 15-min simulations at 50 l·m^2^·h intensity (selected according to rainfall record in the study area from 2000 to 2013) in consecutive weeks, allowing the substrate to dry in between. We visually checked thallus detachment after each simulation, removing detached thalli to avoid interference on the following check. Once the substrate dried after the second simulation, we assessed thallus attachment by manual inspection applying gentle sideways pressure on the thalli to verify whether the thalli were attached or moved without resistance. We measured CO_2_ exchange as an indicator of thallus vitality after adhesive application^38^,^39^. The thalli used in the rainfall simulation were left in their trays outdoors for two months (May-June) under ambient conditions. The CO_2_ exchange was measured with an infrared gas analyser (EGM-4 Environmental Gas Monitor and a SRC-1 Soil Respiration Chamber, PP systems, Hitchin, U.K.). Six thalli per tray (30 per treatment) were moistened and placed together inside the chamber (to improve CO_2_ detection) on a sterile plastic surface (to avoid substrate interference in the measurements). Five measurements per treatment were performed by alternating treatments. Measurements were taken between 10:00 and 13:00 h (local time, GMT + 1). This period is considered representative of daily averages of CO_2_ exchange for these lichens^40^,^41^.

#### Field experiment

The experiment was set up in March 2014 on a conditioned flat area consisting of bare gypsum spoil generated from gypsum quarrying. A total of 30 permanent plots of 0.5 × 0.5 m were established with a randomised design, including 5 replicate plots per each of the 6 adhesive treatments. In the centre of each plot (Fig. 1d), 35 thalli were positioned using a 50 × 50 cm quadrat with a 5 × 5 cm grid (5 replicate plots × 6 treatments × 35 thalli = 1050 thalli). Each thallus was transferred and fixed to the centre of a grid square with the corresponding plot adhesive applying gentle pressure to attach it, and its position was identified in order to record thallus detachment over time. Thalli were fixed by applying an adhesive treatment (2 ml with a 200-ml syringe) to the lower surface of the thallus, except for the water treatment, which was applied by spraying tap water (~200 ml) to moisten the plot substrate. We monitored lichens visually for thallus detachment over a 15-month period (March 2014 to May 2015), weekly during the first three months and monthly afterwards. Detached thalli were removed to avoid interference on the following sampling dates. Additionally, we measured chlorophyll fluorescence using the ratio F_v_/F_m_ as an indicator of lichen vitality 16 months (June, 2015) after the application of the adhesives. Chlorophyll fluorescence is a sensitive indicator of photosystem II (PSII) efficiency, used to detect stress and to assess lichen vitality^39^,^42^,^43^. Fluorescence was measured on 10 thalli (when available) per replicate plot with a chlorophyll fluorimeter (Handy PEA, Hansatech Instruments, Norfolk, U.K.) on soaked (with a spray of water) and dark-adapted (measured by night) samples, applying a saturating flash of light of 3580 μmol s^−1^ m^−2^ for 1 s. As a reference of vital lichens, we included 50 15-mm thalli prepared with fresh material collected from the undisturbed habitat (Hab) two hours before the measurements were taken following the same procedure, and we evaluated the efficiency of PSII by measuring the F_v_/F_m_ ratio^44^.

#### Data analyses

We assessed the effect of adhesives on thallus attachment over time using the Kaplan-Meier log-rank survival analysis (R “survival” package^45^) and fitting mixed-effects Cox proportional hazard models (R “coxme” package^46^) using trays or quadrats as random factors, respectively, in rain-simulation or field experiments. The effect of adhesives on CO_2_ exchange in rainfall simulations was evaluated by fitting generalised linear models (GLMs) with a Poisson error distribution and log link function (R “stats” package^47^). We tested for the effect of adhesives (i.e. fixed factor) on fluorescence yield in the field experiment, fitting generalised linear mixed models (GLMMs) with a binomial error distribution and logit link function, using quadrats as random factors (R “lme4” package^48^). Model parameters were estimated using the Laplace approximation of likelihood. Pairwise multiple comparisons with Tukey’s correction (R “multcomp” package^49^ were made to estimate differences between adhesive treatments. All statistical analyses were performed using R version 3.2.2^47^.

## Results

### Rainfall-simulation experiment

**Figure 2:**
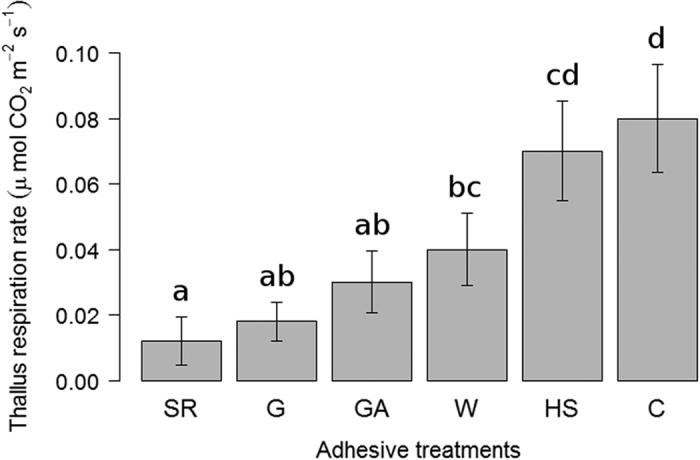
Thallus respiration rate (mean values ± SE) for each adhesive treatment in rain simulations. Treatments: SR, synthetic resin; G, white glue; GA, gum arabic; W, water; HS, hydroseeding; C, control. Different letters represent statistically significant differences between adhesives (p < 0.05).

Rain simulation showed 100% attachment in G, W, and GA and very high for the rest of treatments at the end of the experiment (SR, 98%; HS, 94% and C, 82%; Table 1; Supplementary Fig. S1). As expected, treatment C recorded the lowest attachment, although the post hoc analysis indicated no differences with respect to the other treatments. All treatments had lower detachment risk than did C (Table 1, Cox regression). Thalli detached during the first simulation only in treatments C (12% detachment) and HS (2%), and no detachment was found during the second one. Manual inspection after the substrate dried showed additional detachment in treatments C (6% detachment), HS (4%), and SR (2%). Remarkably, the remaining thalli on the C treatment were attached at the end of this experiment. The analysis of CO_2_-exchange measurements taken on thalli detached for this purpose at the end of the experiment showed significant differences between adhesives, with higher respiration rates in C, HS, W, GA, G, and SR in descending order (Fig. 2, Supplementary Table S3).

### Field-experiment

**Figure 3:**
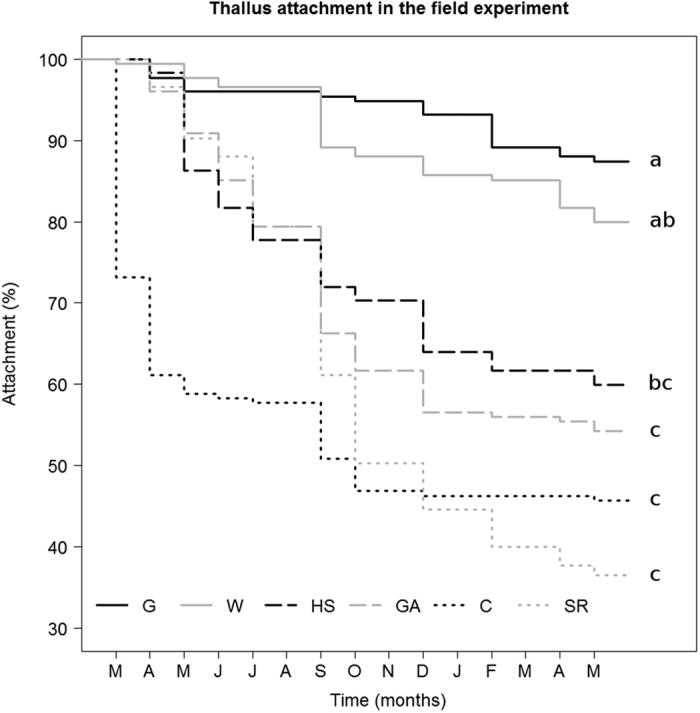
Kaplan-Meier survival curves representing thallus attachment for each adhesive treatment from March 2014 to May 2015 in the field experiment. Treatments: G, white glue; W, water; HS, hydroseeding; GA, gum arabic; C, control; SR, synthetic resin. Different letters at the end of curves represent statistically significant differences between adhesives (p < 0.05).

**Figure 4:**
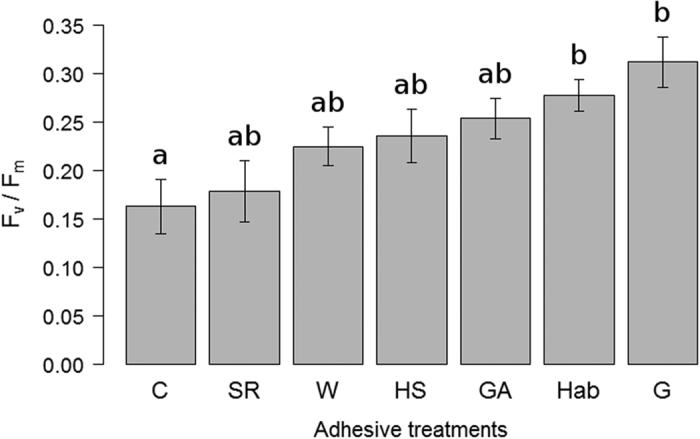
Maximum quantum yield (mean values ± SE) of PSII photochemistry (F_v_/F_m_) in thalli of the lichen *D. diacapsis* 16 months after translocation to gypsum spoils using adhesive treatments: G, white glue; W, water; HS, hydroseeding; GA, gum arabic; C, control; SR, synthetic resin. Thalli from the undisturbed habitat (Hab) were transferred to the same substrate before measurements as a reference. Different letters represent statistically significant differences between adhesives (p < 0.05).

After a 15-month follow-up of the field experiment, our results showed that the adhesive treatment significantly affected thallus attachment (Fig. 3; Table 2). Responses varied depending on the adhesive, with better results in G (87.4% attachment), W (80%), HS (59.9%), GA (54.3%), C (45.7%), and SR (36.6%) in this order, with significant differences between G and W compared to the rest (Table 2). Kaplan-Meier curves showed only moderate detachment in G and W and significantly greater in the rest of the treatments (Fig. 3). Treatment C plummeted in the first month, followed by steady detachment until declining almost completely towards the eighth month. Detachment was more gradual in HS, GA, and SR, with the first two listed treatments with little detachment from the tenth month onwards, and the latter falling steadily until the end of the follow-up, even underperforming the C treatment (Fig. 3). The Cox proportional hazard analysis also showed significant differences between treatments. All treatments reduced the risk of detachment compared to the control, except SR, which presented a similar risk (Table 2, Cox regression). The photosynthetic activity as F_v_/F_m_ for the treatments was C (0.16), SR (0.18), W (0.22), HS (0.24), GA (0.25), Hab (0.28) and G (0.31), with significant differences only between C, and G, and Hab (Fig. 4, Supplementary Table S4).

## Discussion

Our findings indicated that white glue, water, hydroseeding stabiliser and gum arabic improved the attachment of *D. diacapsis* thalli, although they also altered lichen vitality. Remarkably, the water treatment (i.e. wetting the spoils) significantly improved thallus attachment without compromising their vitality, which would be especially advantageous to optimise crust material in translocation actions.

Thallus attachment to the substrate was similar for all adhesives in rainfall simulations. Although the thalli were exposed to considerable rain intensity and inclination in this experiment, the level, angle or time exposed to these factors were not sufficient to show differences between treatments. By contrast, thallus attachment differed depending on the adhesive when exposed to field conditions over a longer period (differences were noticeable from the first month onwards). The difference between experiments appears to be explained by the greater exposure time in the field to rain and wind. These factors can encourage lichen dispersal in open areas^4^,^21^, but for the same reason they can also make it difficult for thalli to remain at the same specific site, hindering establishment. In our field experiment, we found thallus attachment using white glue and water was greatest and remarkably better than for the other treatments throughout the follow-up. By contrast, the performance of hydroseeding stabiliser and gum arabic was inferior and similar to the control towards the end of the experiment. Although the attachment achieved with the synthetic resin was similar to that using the hydroseeding stabiliser and gum arabic for nearly 8 months, it lost its adhesive properties and eventually registered the lowest results.

Our results suggest that water followed by substrate drying played a central role in thallus attachment. Although we assumed *a priori* that making the spoils wet would have an antagonistic effect on thallus attachment, the opposite happened in both the rainfall-simulation and field experiments. In rainfall simulations, most of the thalli in all treatments were attached to the substrate after it dried, this being especially remarkable in the control treatment, where no adhesive had been applied, but where water acted as such after the first simulation. This pattern was observed in the field also. Most thalli in the control treatment detached in the first two months but stabilised afterwards despite heavy rain in the following months. In fact, all treatments seemed to stabilise except the synthetic resin. A possible explanation is that the synthetic resin is a hydrophobic adhesive and remained on the thalli, preventing them from reattaching. While water may weaken the relationship between thalli and the substrate during a rain event, it also helps to create some bonds with the substrate surface^18^,^50^. The surface becomes sticky due to the high cohesiveness of silt and clay particles when mixed with moderate amount of water. Then the drying process makes these bonds more stable, helping lichens to attach. This pattern has been reported in nature (e.g. with seeds^51^ or soil particles^52^) and, as our results reveal, it is one of the ways lichen propagules create bonds and attach to certain substrates.

Another key issue is how the different treatments affect thallus vitality. A given adhesive could aid thallus attachment, but if significantly harmed the lichen’s physiology, then use of the product would be unacceptable. In this regard, vitality measurements determined that some of the treatments tested reduced thallus activity, although no one was so aggressive as to completely inhibit respiration or photosynthetic processes. The results of CO_2_ exchange showed thallus activity in rainfall simulations to be reduced by the adhesive applied. The highest respiration rate was registered in the control treatment, followed by the hydroseeding stabiliser, water, and the rest of treatments. Contrary to expectations, the water treatment differed from control despite that thalli in both treatments were treated only with water (prior or during the rainfall simulation). Although the CO_2_ exchange was not as high as in the control treatment, thalli treated with water were active and there is no apparent reason why water would reduce their vitality, but rather the opposite. In the field experiment, the efficiency of PSII (F_v_/F_m_) used as stress indicator^53^ showed no differences between the thalli translocated using adhesives and those freshly collected in the undisturbed habitat. Although no significant differences were found between treatments, with the use of the synthetic resin, thalli showed reduced activity and some signs of necrosis, and thus this adhesive is not recommendable. The control treatment recorded the lowest value, probably due to the lowest attachment to the substrate. The activity for all the other adhesives was between 0.22 and 0.31, and the activity for thalli in the habitat (Hab) used as reference was within this range (0.28). These values are similar to PSII efficiency on undisturbed north and south-facing populations of *D. diacapsis* studied in Pintado *et al*.^54^, estimated, respectively, as 0.25 and 0.28 by Maestre *et al*.^3^. Thus, we can infer that translocation onto the gypsum spoil using most of the adhesives studied here was not so stressful as to alter the photosynthetic process and make the F_v_/F_m_ differ from nature.

Therefore, our results suggest that translocation can help some components of the BSCs to recover. We have demonstrated that *D. diacapsis* is amenable to translocation and can be moved from its habitat onto gypsum spoils, but this methodology could be extended to other gypsum-crust components in substrates having similar properties (i.e. disturbed original habitat or topsoil used for restoration). More than 50% of the lichen species in gypsum habitats have similar morphology and ecology^4^ and the use of this methodology with them is especially appealing. In addition, other terricolous lichen communities on similarly performing substrates in other drylands could benefit from this approach. The crust material can be salvaged from donor habitats prior quarrying. Complete thalli or fragments can be scalped from the parent material along with other BSCs species^14^,^15^. This material could be applied directly or stored for relatively long periods providing some flexibility for translocation actions^10^,^14^. The recipient substrate can be sprayed with water to create a sticky surface to receive the crust material, and then let the substrate dry until the thalli bind, as our results reveal.

Areas meeting the environmental requirements that define the occurrence of these crusts should be selected for translocation^24^. Their occurrence has been related to variables such as substrate stability^4^,^55^, orientation and water availability^54^, soil respiration^55^, and complex relations with the surrounding vegetation^3^,^55^,^56^,^57^. Accordingly, Martínez *et al*.^55^ reported that high bare-soil cover and low plant-litter cover were positively related to the presence of gypsum crustose lichens. While this scenario is frequent right away after quarrying, invasive early successional plant species (e.g. *Dittrichia viscosa, Pitptatherum milliaceum, Moricandia arvensis,* in our area) could strongly reduce open areas available for lichens over the short term. Thus, where possible, it would be advisable to translocate lichens to plant interspaces in areas where gypsum vegetation had already developed (naturally or restored) aiding it to escape early successional competitors. Additionally, these areas would provide a gradient of conditions for translocation, depending on crust species-specific requirements related to plant proximity (shade, moisture, organic matter, etc.^55^,^56^,^57^,^58^. Other components of gypsum BSCs (i.e. cyanobacteria and algae) have been reported to develop comparatively fast through colonisation^15^ or inoculation^14^,^15^,^16^. Thus, a combination of translocation with additional inoculation measures^16^ or the use of already colonised substrates (i.e. restored gypsum spoils, topsoil, disturbed original habitat) could help facilitate certain limiting processes (e.g. contact between mycobiont spores and photobionts^59^) and promote a more complete recovery of gypsum BSCs.

In conclusion, here we have shown that the use of certain adhesives can significantly increase lichen attachment to the substrate without compromising their vitality. Particularly, our study shows the central role that water and the subsequent drying of the substrate play for thallus attachment, and offers a methodology with implications for restoration of disturbed gypsum BSCs. This study represents one of the first studies to evaluate the translocation of lichens in gypsum drylands and the first to test adhesives for lichen attachment onto gypsum quarry spoils. This methodology needs to be tested over time using other species and considering disturbances in gypsum habitats. Further protocols should be designed, assessing the ecological, technical, and economic viability of translocation, in order to confirm their applicability to large-scale restoration of gypsum BSCs. The results in the present study are encouraging and open an opportunity to accelerate the recovery of BSCs in disturbed gypsum habitats.

## Additional Information

**How to cite this article:** Ballesteros, M. *et al*. Successful lichen translocation on disturbed gypsum areas: A test with adhesives to promote the recovery of biological soil crusts. *Sci. Rep.* **7**, 45606; doi: 10.1038/srep45606 (2017).

**Publisher's note:** Springer Nature remains neutral with regard to jurisdictional claims in published maps and institutional affiliations.

## Supplementary Material

Supplementary Information

## Figures and Tables

**Table 1:** Thallus attachment (%) and results of the Cox proportional-hazard regression for adhesive treatments in the rainfall-simulation experiment (control treatment established as reference).

Treatment	Attachment (%)	Cox proportional-hazard regression				
		HR	Estimate	SE	z	p
Synthetic resin	98	0.10	−2.292	1.096	−2.09	**0.037**
Hydroseeding	94	0.31	−1178	0.731	−1.61	0.110
Control	82	1	—	—	—	—
		Random effects: Variance: 0.222; SD: 0.472				

Treatments with 100% attachment (white glue, water, gum arabic) are not included in the analysis. HR: Hazard ratio <1 means thalli using this adhesive treatment had a lower risk of detachment than did control. Results with p < 0.05 are in bold.

**Table 2:** Thallus attachment (%) and results of Cox proportional-hazard regression for adhesive treatments in the field experiment (control treatment established as reference).

Treatment	Attachment (%)	Cox proportional-hazard regression				
		HR	Estimate	SE	z	p
White glue	87.4	0.13	−2.024	0.365	−5.55	<**0.001**
Water	80	0.22	−1.528	0.341	−4.48	<**0.001**
Hydroseeding	59.9	0.52	−0.669	0.320	−2.09	**0.04**
Gum arabic	54.3	0.62	−0.482	0.315	−1.53	0.13
Control	45.7	1	—	—	—	—
Synthetic resin	36.6	0.93	−0.073	0.310	−0.24	0.81
		Random effects: Variance: 0.190; SD: 0.436				

HR: Hazard ratio <1 means thalli using this adhesive treatment had a lower risk of detachment than did control. Results with p < 0.05 are in bold.
