# Supplementary material for: Successful lichen translocation on disturbed gypsum areas: A test with adhesives to promote the recovery of biological soil crusts
Source: Sci Rep. 2017 Apr 3;7:45606. doi: 10.1038/srep45606 (PMC5377308; doi:10.1038/srep45606)
Supplement: Supplementary Information [file srep45606-s1.pdf]

Supplementary Information for:

Successful lichen translocation on disturbed gypsum areas: A test with adhesives to promote the recovery of biological soil crusts

Ballesteros, M.[1,*], Ayerbe, J.[1], Casares, M.[1], Cañadas, E.M.[1], Lorite, J.[1,2]

[1] Department of Botany, Faculty of Sciences, University of Granada, Campus de Fuentenueva s/n, 18071, Granada, Spain

[2] iEcolab. Interuniversitary Institute for Earth System Research (IISTA) - University of Granada, Av. del Mediterráneo, 18006, Granada, Spain

Correspondence and requests for materials should be addressed to M.B (miguelballesterosjimenez@gmail.com; ballesteros@ugr.es).

Supplementary Table S1. Cover (%) of lichens and number of species (mean ± SE) determined on 18 soil cores of 25 x 25 cm and 10 cm depth collected in the study area (Ibarz, 2012).

	Mean ± SE
Total lichen cover per soil core (%)	76.8 ± 4.3
Number of species	9.1 ± 0.6
Individual species cover (%)	
Acarospora nodulosa var. *reagens* (Zahlbr.) Clauzade & Cl. Roux	2.1 ± 0.7
Acarospora placodiiformis H. Magn.	7.3 ± 1.8
Buellia zoharyi Galun	1.0 ± 1.0
Cladonia foliacea (Huds.) Willd	0.1 ± 0.1
Collema sp.	4.3 ± 1.2
Diploschistes diacapsis (Ach.) Lumbsch	54.7 ± 5.4
Fulgensia desertorum (Tomin) Poelt	0.9 ± 0.5
Fulgensia fulgens (Sw.) Elenkin	6.3 ± 1.8
Fulgensia poeltii Llimona	0.8 ± 0.4
Fulgensia subbracteata (Nyl.) Poelt	7.8 ± 2.1
Psora albilabra (Dufour) Körber	0.6 ± 0.3
Psora decipiens (Hedw.) Hoffm.	7.4 ± 2.8
Psora saviczii (Tomin) Follmann & Crespo	1.0 ± 0.3
Squamarina cartilaginea (With.) P. James	1.9 ± 0.6
Squamarina lentigera (Weber) Poelt	6.8 ± 2.6
Toninia sedifolia (Scop.) Timdal	3.8 ± 1.5

Ibarz, N., 2012. Primera experiencia de restauración de la costra liquénica en ambientes semiáridos. Master thesis (unpublished). Universidad de Granada.

Supplementary Table S2. Mean values (± standard deviation) of the physicochemical characterization of the gypsum spoil used (Aguilera, 2012). Twelve gypsum spoil samples were randomly collected in the study site at 0-30 cm depth to determine the substrate properties. N is the number of samples used for the analyses. The analyses were conducted following the methodology in Mañares et al. (1998) and MAPA (1994). [a] Exchangeable cations.

Variable	N	Gypsum spoil
Gravel (>2mm) (%)	4	33.48 ± 3.78
Sand (2-0.05 mm) (%)	12	8.99 ± 2.17
Coarse silt (0.05-0.02 mm) (%)	12	9.60 ± 6.22
Fine silt (0.02 mm) (%)	12	41.44 ± 7.60
Clay (<0.02 mm) (%)	12	39.97 ± 8.22
pH	12	7.79 ± 0.04
Cation exchange capacity ($cmol_+$/kg)	12	8.15 ± 1.77
Ca^{2+} ($cmol_+$/kg)[a]	12	7.68 ± 1.83
Mg^{2+} ($cmol_+$/kg)[a]	12	0.22 ± 0.06
Na^+ ($cmol_+$/kg)[a]	12	0.04 ± 0.01
K^+ ($cmol_+$/kg)[a]	12	0.20 ± 0.07
Total carbon (%)	12	3.26 ± 0.45
Inorganic carbon (%)	12	3.27 ± 0.42
Organic carbon (%)	7	0.04 ± 0.03
Total N (%)	12	0.029 ± 0.005
$CaCO_3$ (%)	12	27.25 ± 3.52
Gypsum (%)	12	47.96 ± 28.22
Electrical conductivity (dS/·m)	12	2.27 ± 0.01
Water retention at field capacity (%)	8	31.09 ± 1.18
Water retention at wilting point (%)	8	20.98 ± 0.96
Available-water content (%)	8	10.11 ± 1.01

Aguilera, R., 2012. Evaluación de distintas técnicas de restauración ecológica en taludes de canteras de yesos. Master thesis (unpublished). Universidad de Granada.

Mañares, A., Sánchez, J., de Haro, S., Sánchez, S.T., del Moral, F. *Análisis de Suelos, Metodología e Interpretación.* (Servicio de Publicaciones Universidad de Almería, Almería, 1998).

MAPA. *Métodos Oficiales de Análisis. Tomo III.* (Secretaría General Técnica del Ministerio de Agricultura, Pesca y Alimentación (MAPA), 1994).

Supplementary Table S3. Effect of adhesive treatment on thallus respiration rate (CO_2 exchange) in the rainfall-simulation experiment, evaluated fitting a generalised linear model (GLM). Results with $p < 0.05$ are in bold.

Treatment	Estimate	SE	z	p
Intercept	-2.369	0.131	-18.044	**<0.001**
White glue	-1.496	0.307	-4.874	**<0.001**
Water	-0.659	0.225	-2.931	**0.003**
Hydroseeding	-0.148	0.193	-0.769	0.442
Arabic gum	-1.016	0.255	-3.989	**<0.001**
Synthetic resin	-1.663	0.329	-5.055	**<0.001**

Supplementary Table S4. Effect of adhesive treatment on maximum quantum yield of PSII photochemistry (F_v/F_m), evaluated fitting a generalised linear mixed model (GLMM). Adhesive treatment as fixed factor and quadrat as random factor. Results with $p < 0.05$ are in bold.

Treatment	Estimate	SE	z	p
Intercept	-1.75	0.143	-12.203	**<0.001**
Synthetic resin	0.111	0.215	0.514	0.607
Water	0.341	0.163	2.094	0.036
Hydroseeding	0.400	0.171	2.344	0.019
Arabic gum	0.350	0.168	2.079	0.038
Habitat	0.564	0.152	3.722	**<0.001**
White glue	0.596	0.159	3.758	**<0.001**
Random effects: Variance: 0.004; SE: 0.064				

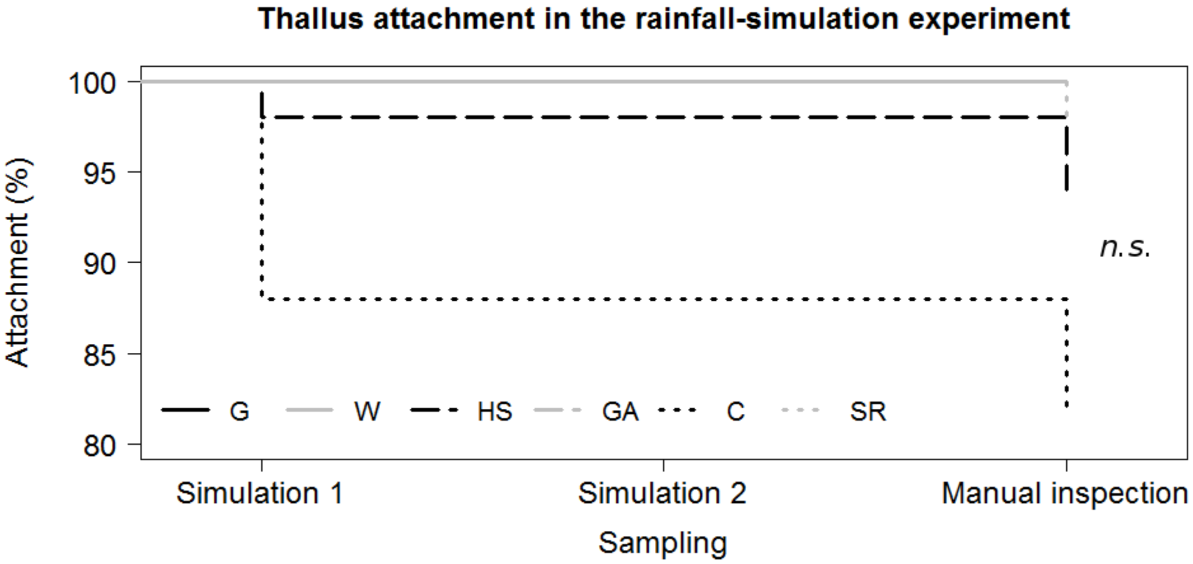

Supplementary Figure S1. Kaplan-Meier survival curves representing thallus attachment for each adhesive treatment after two simulated rainfall events and manual inspection once the substrate dried. Note treatments with white glue, water, and gum arabic had 100% attachment. Treatments: G, white glue; W, water; HS, hydroseeding; GA, gum arabic; C, control; SR, synthetic resin. There were no significant differences between adhesives at $p < 0.05$.
